# Deubiquitinases as pivotal regulators of T cell functions

**DOI:** 10.1007/s11684-018-0651-y

**Published:** 2018-07-27

**Authors:** Xiao-Dong Yang, Shao-Cong Sun

**Affiliations:** 1 Shanghai Institute of Immunology, Shanghai Jiao Tong University School of Medicine, Shanghai 200025, China; 2 Department of Immunology, The University of Texas MD Anderson Cancer Center, 7455 Fannin Street, Box 902, Houston, TX 77030, USA; 3 The University of Texas Graduate School of Biomedical Sciences at Houston, Houston, TX 77030, USA

**Keywords:** deubiquitinase, ubiquitination, T cell activation, T cell differentiation, T cell tolerance

## Abstract

T cells efficiently respond to foreign antigens to mediate immune responses against infections but are tolerant to self-tissues. Defect in T cell activation is associated with severe immune deficiencies, whereas aberrant T cell activation contributes to the pathogenesis of diverse autoimmune and inflammatory diseases. An emerging mechanism that regulates T cell activation and tolerance is ubiquitination, a reversible process of protein modification that is counter-regulated by ubiquitinating enzymes and deubiquitinases (DUBs). DUBs are isopeptidases that cleave polyubiquitin chains and remove ubiquitin from target proteins, thereby controlling the magnitude and duration of ubiquitin signaling. It is now well recognized that DUBs are crucial regulators of T cell responses and serve as potential therapeutic targets for manipulating immune responses in the treatment of immunological disorders and cancer. This review will discuss the recent progresses regarding the functions of DUBs in T cells.

## Introduction

T cells serve as a central player in adaptive immunity against infections and oncogenesis. Following their development in the thymus, naïve CD4^+^ and CD8^+^ T cells migrate to the periphery and circulate between the blood and peripheral lymphoid organs. Upon encountering a foreign antigen, naïve T cells are activated to undergo proliferation and differentiation, generating different subsets of effector T cells that are required for pathogen destruction [1,2]. Under normal situations, T cells efficiently respond to foreign antigens but are tolerant to self-antigens, thereby preventing the development of autoimmune diseases [3]. T cell tolerance is induced along with T cell development in the thymus (central tolerance) or in the periphery through induction of T cell anergy and the immunosuppressive action of regulatory T (Treg) cells [4].

The activation of naïve T cells is initiated by engagement of the T cell receptor (TCR) with a cognate antigen presented by antigen-presenting cells (APCs) and also requires the simultaneous ligation of costimulatory receptors, particularly CD28, by specific ligands on APCs [1]. Upon activation, CD4^+^ T cells differentiate into T helper (Th) cells, including Th1, Th2, Th17, and T follicular helper (Tfh) cells, as well as the inducible regulatory T (iTreg) cells [2,5]. The different subsets of Th cells produce different cytokines and mediate distinct effector functions. Th1 cells express the cytokine interferon γ (IFNγ) and activate macrophages for destruction of intracellular pathogens; Th2 cells express IL-4 and related cytokines and are important for immune responses against extracellular parasites; Th17 cells produce the signature cytokines IL-17A and IL-17F and mediate immune responses against extracellular pathogens; and Tfh cells play a crucial role in humoral immunity by facilitating the activation and differentiation of B cells and the formation of germinal centers [2,5,6]. In addition to their normal functions in immune responses, aberrantly activated CD4^+^ T cells, particularly Th1 and Th17 cells, are also involved in the pathogenesis of autoimmune and inflammatory diseases [7]. The CD8^+^ effector T cells, called cytotoxic T lymphocytes (CTLs), are specialized for direct lysis of target cells infected with viruses and certain bacteria [8,9]. CD8^+^ T cells also attack cancer cells and play a primary role in mediating anticancer immunity, although CD4^+^ T cells facilitate the generation and maintenance of CD8^+^ effector T cells through cytokine production and activation of APCs [10,11].

Signal transduction from the TCR and the costimulatory receptor CD28 plays a primary role in regulating the development, activation, and differentiation of T cells [12]. In addition to the antigen-recognition subunits (α and β), the TCR complex contains several signaling subunits, including ζ, CD3δ, CD3γ, and CD3ε chains [1]. The ζ and CD3 chains contain immunoreceptor typrosine-based activation motifs (ITAMs), which are crucial for TCR signaling. Ligation of TCR and CD28 elicits TCR-proximal signaling events, including activation of the Src kinase Lck, which phosphorylates conserved typrosine residues of the ζ chain ITAMs to create binding sites for recruiting the tyrosine kinase Zap70 [1]. Upon activation by Lck in the TCR complex, Zap70 phosphorylates a number of target proteins, including the scaffold proteins LAT and SLP76 (also known as Lcp2), thereby amplifying the TCR/CD28 signal by triggering activation of multiple signaling pathways, including the calcium, protein kinase C θ (PKCθ), and RAS pathways. Consequently, the TCR signaling triggers activation of various downstream pathways including those leading to the activation of three major families of transcription factors, NF-κB, AP1, and NFAT [1]. Under normal conditions, the TCR/CD28 signaling is tightly controlled by negative regulators, which serves as an important mechanism to prevent aberrant T cell responses and to maintain T cell tolerance to self-tissues [3]. Defect in TCR-negative regulatory mechanisms contributes to the development of autoimmunity and inflammatory diseases [2,13].

**Fig. 1:**
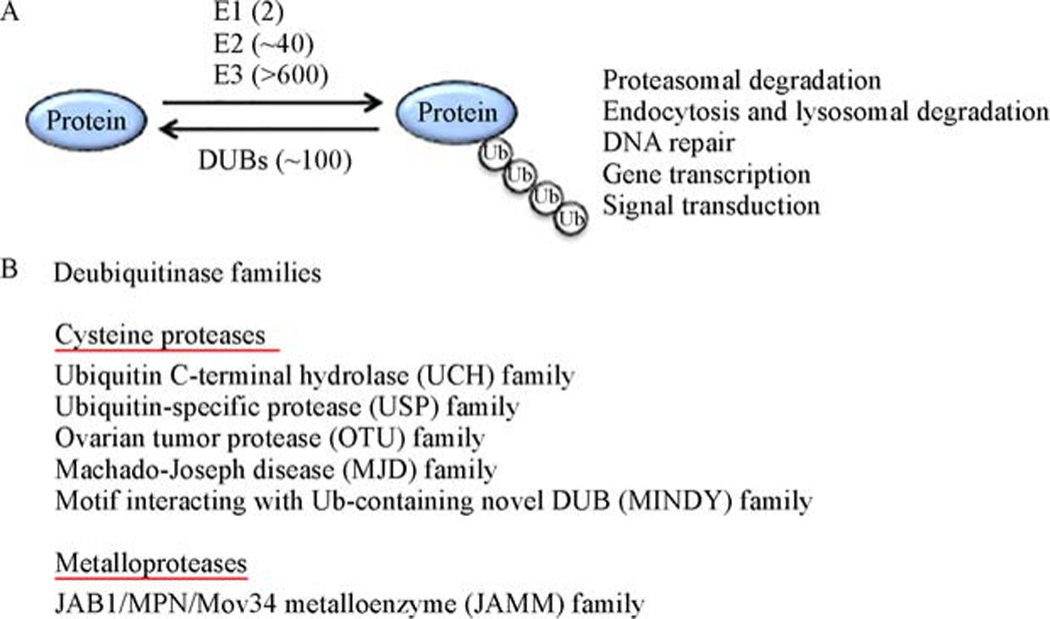
Ubiquitination is a reversible reaction counter regulated by ubiquitinating enzymes and DUBs. (A) Ubiquitin conjugation onto a target protein is catalyzed by the sequential action of three ubiquitinating enzymes, E1, E2, and E3. Mammalian cells have 2 E1s, about 40 E2s, and more than 600 E3s. E3s mediate substrate recognition and determine the specificity of protein ubiquitination. Ubiquitination can occur via formation of different types of ubiquitin chains and regulate diverse cellular functions. Deubiquitinases (DUBs) cleave ubiquitin chains and deconjugate ubiquitin from substrates, thereby reversing the ubiquitination reaction. (B) DUBs are classified into six families, including five families of cysteine proteases and one family of metalloprotease.

An important mechanism that regulates TCR signaling is ubiquitination, a posttranslational mechanism of protein modification that involves covalent conjugation of monoubiquitin or poly-ubiquitin chains onto lysine (K) residues of target proteins [14]. Ubiquitination is catalyzed by the sequential action of a ubiquitin-activating enzyme (E1), ubiquitin-conjugating enzymes (E2), and ubiquitin ligases (E3) [14] (Fig. 1). Formation of polyubiquitin chains involves isopeptide bond linkage of the C-terminal glycine residue of a ubiquitin with one of the 7 internal K residues or the N-terminal methionine residue of the preceding ubiquitin, thus creating a large variety of polyubiquitin chains, including K6, K11, K27, K29, K33, K48, K63, and M1 (also called linear) ubiquitin chains [15,16]. The different types of polyubiquitin chains have distinct conformation that can be bound by different ubiquitin binding proteins and, thereby, exert distinct biological functions [15–17]. For example, the K48- and K11-linked ubiquitin chains mediate protein degradation in the proteasome, whereas K63-linked and linear ubiquitin chains are important for signal transduction involved in activation of the transcription factor NF-κB [16–18].

Ubiquitination is a reversible process with the reverse reaction being catalyzed by a family of isopeptidases, known as deubiquitinases (DUBs) (Fig. 1). DUBs can be classified into six families: ubiquitin C-terminal hydrolase (UCH), ubiquitin-specific protease (USP), ovarian tumor (OTU), Machado-Joseph disease (MJD), motif interacting with Ub-containing novel DUB (MINDY), and JAB1/MPN/Mov34 metalloenzyme (JAMM) families [19–21]. The JAMM DUBs are metalloproteases, whereas all the other families are cysteine proteases. DUBs differ in many ways of their modes of functions, including substrate interaction, ubiquitin chain cleavage, and specificity toward different types of ubiquitin chains [21]. During recent years, mouse model studies have demonstrated important roles for DUBs in regulating diverse biological processes including the development, activation, and differentiation of T cells. In addition, DUBs are crucial regulators of T cell tolerance and immune homeostasis. In this review, we will discuss the recent progresses regarding the functions of DUBs in T cells (Table 1).

### DUBs in T cell development

T cells develop and mature in the thymus following sequential developmental stages that can be defined based on surface expression of the T cell coreceptors, CD4 and CD8 [22]. The early CD4^-^CD8^-^ double-negative (DN) stage is characterized by rearrangement of TCRα chain to form a pre-TCR required for signal transduction that drives the progression of DN thymocytes to CD4^+^CD8^+^ double-positive (DP) thymocytes. During the DP stage, thymocytes express functional αβ TCRs, as a result of TCRα gene rearrangement, and undergo positive and negative selections based on detection of self-peptide-MHC complexes displayed on thymic epithelial cells [23]. DP thymocytes expressing TCRs that fail to recognize or bind with high affinity of the self-peptide-MHC complexes are deleted, whereas those with TCR that bind self-peptide-MHC complexes with intermediate affinity are positively selected and further progressed to CD4^+^ or CD8^+^ single-positive (SP) mature thymocytes [22,23]. Thus, the TCR signaling strength plays a crucial role in regulating thymocyte progression from the DP to SP stages.

In addition to phosphorylation, ubiquitination serves as a major mechanism regulating TCR signaling [24]. A number of E3 ubiquitin ligases have been characterized in the regulation of TCR signaling in both thymocytes and peripheral T cells, although role of DUBs in regulating TCR signaling, particularly during thymocyte development, is still poorly understood. The initial evidence for the involvement of DUBs in regulating thymocyte development was obtained from the study of mice carrying a genetic deficiency in CYLD [25], a DUB that was initially discovered as a tumor suppressor associated with cylindromatosis [26]. The CYLD deficiency attenuates thymocyte development from the DP to SP stages, causing reduced T cell numbers in the peripheral lymphoid organs [25]. CYLD targets the protein tyrosine kinase LCK and plays an important role in mediating TCR signaling during the transition of DP thymocytes to mature SP thymocytes [25]. Moreover, the role of CYLD in regulating thymocyte development may also involve regulation of IKK activation [27]. In addition to its cell-intrinsic function in thymocyte development, CYLD regulates the differentiation of medullary thymic epithelial cells (mTECs) that are required for thymocyte negative selection [28].

CYLD also has a crucial role in regulating the development of natural killer T (NKT) cells [29], a family of innate-like T cells responding to lipid antigens and regulating diverse aspects of immune and autoimmune responses [30,31]. NKT cells are developed in the thymus, where they originate from CD4^+^CD8^+^ DP thymocytes expressing a semi-invariant TCR [30,32]. NKT cell development involves an early stage of positive selection mediated by recognition of self-lipid antigens presented by an MHCI-like molecule, CD1d, and subsequent maturation following progressive stages [30]. CYLD is dispensable for NKT cell maturation but is required for the survival of immature NKT cells. CYLD deficiency attenuates NKT cell signaling stimulated by the survival cytokine IL-7, which in turn is due to aberrant activation of the transcription factor NF-κB [29]. Precisely how NF-κB activation contributes to impaired IL-7 receptor (IL-7R) signaling is unclear, but this involves downregulation of the α chain of IL-7R (IL-7Rα). The development of NKT is also subject to regulation by the DUB A20. In contrast to CYLD, A20 is not important for survival of immature NKT cells but plays a crucial role in mediating NKT cell maturation [33]. T cell-specific A20 deficiency greatly reduces the number of mature NKT cells without affecting the early stages of immature NKT cells. Based on cytokine secretion profiles, NKT cells are divided into NKT1, NKT2, and NKT17 subsets, characterized by production of IFNγ, IL-4, and IL-17, respectively [34]. A20 deficiency reduces the number of NKT1 and NKT2 cells in the peripheral blood and organs without affecting NKT17 cells [33]. It appears that the A20-deficient NKT1 and NKT2 cells are aberrantly activated and targeted for activation-induced cell death [33].

Another DUB that regulates T cell development is USP8, which is characterized by the presence of two atypical SH3 binding motifs and a 14–3-3 binding motif [35]. In response to TCR/CD28 stimulation, USP8 interacts with the signaling adaptor Gads and 14–3-3b in the TCR signalosome and becomes cleaved by caspases. T cell-specific USP8 deficiency in mice has no effect on the production of immature DN and DP thymocytes but greatly reduces the number of SP thymocytes, suggesting an essential role for USP8 in regulating thymocyte maturation [35]. Interestingly, USP8 is dispensable for TCR signaling but is required for IL-7Ra gene induction by the transcription factor Foxo1 [35]. The reduced level of IL-7R may contribute to impaired thymocyte maturation in the USP8-deficient mice. A more recent study suggests that during thymocyte positive selection, USP8 interacts with and stabilizes CHMP5, an ESCRT (endosomal-sorting-complex-required-for-transport) protein required for thymocyte post-selection survival and maturation [36]. Thus, the function of USP8 in regulating T cell development may involve different mechanisms. Given the crucial role of ubiquitination in the regulation of TCR and cytokine receptor signaling, it is anticipated that more DUBs will be characterized in the regulation of T cell development.

### DUBs in T cell activation and survival

**Fig. 2:**
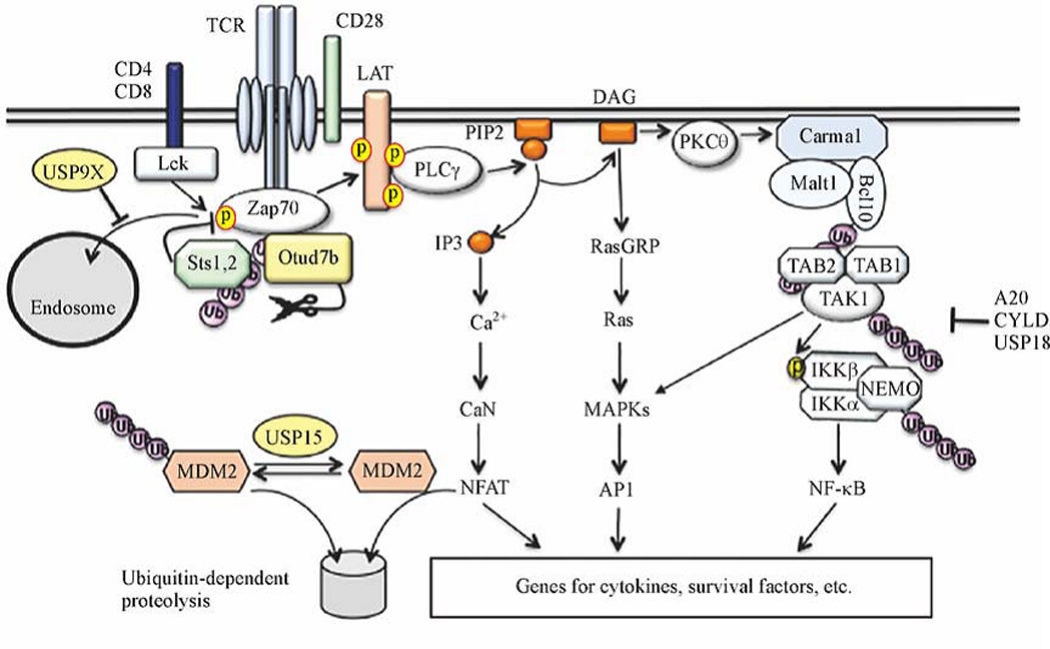
DUBs regulating TCR signaling. DUBs regulate both TCR-proximal and downstream signaling events. Otud7b deconjugates nondegradative ubiquitin chains from Zap70 to prevent its association with a negative-regulatory phosphatase, Sts1 or Sts2, thereby promoting Zap70 activation. USP9X deubiquitinates Zap70 to prevent endosome sequestration of ubiquitinated Zap70. USP15 deubiquitinates and stabilizes MDM2, an E3 ligase mediating ubiquitination and proteolysis of an NFAT family member, NFATc2, and negatively regulating TCR signaling. Several DUBs, including A20, CYLD, and USP18, deconjugate K63-linked ubiquitin chains from the TAK1/IKK signaling complex to negatively regulate this signaling pathway.

Ubiquitination plays a critical role in the regulation of TCR/CD28 signaling in both T cell homeostasis and activation [24]. A well-characterized function of ubiquitination is to regulate TCR/CD28-stimulated activation of TAK1 and its downstream kinases IKK and JNK. This signaling axis requires an intermediate signaling complex, composed of the scaffold protein CARMA1, the adaptor Bcl-10, and the paracaspase MALT1 [37]. In response to the TCR/CD28 signal, this so-called CBM complex stimulates conjugation of K63-linked polyubiuquitin chains onto a number of signaling components, including Bcl-10, MALT1, TAK1, and the IKK regulatory subunit NEMO, which promotes assembly of the TAK1 signaling complex and the catalytic activation of TAK1 and its targets, IKK and JNK [24] (Fig. 2). TAK1 is associated with two regulatory subunits, TAB1 and TAB2, the latter of which possesses a ubiquitin-association (UBA) domain capable of binding K63-linked ubiquitinations [37]. It has been proposed that TAB2-mediated ubiquitin binding serves as a trigger for TAK1 activation. However, strong evidence suggests that direct conjugation of ubiquitin chains to TAK1 also contributes to its catalytic activation [38,39].

Under homeostatic conditions, TAK1 undergoes dynamic ubiquitination in T cells, which is controlled by the DUB CYLD [39] (Fig. 2). CYLD deficiency results in hyper-ubiquitination and activation of TAK1, causing spontaneous activation of IKK and JNK as well as the downstream transcription factor NF-κB [39]. Consistently, the CYLD-deficient mice have perturbed T cell homeostasis, characterized by a profound increase in the frequency of T cells with activated phenotype. When adoptively transferred to Rag1-KO mice, the CYLD-deficient T cells are hyper-responsive to commensal antigens and cause severe colitis. These mutant T cells are also hyper-responsive to *in vitro* activation by CD3 and CD28 agonistic antibodies for proliferation and cytokine projection. Thus, CYLD is a crucial negative regulator of TCR activation and homeostasis. In line with these findings, a recent study demonstrates that the CYLD deficiency promotes CD8^+^ T cell responses and renders mice more resistant to experimental cerebral malaria (ECM) induction in a murine model [40]. Like CYLD, USP18 targets the ubiquitin-dependent kinase TAK1. It appears that CYLD is more important for controlling the ubiquitination and signaling function of TAK1 under homeostatic conditions [39], whereas USP18 inhibits TCR-stimulated TAK1 ubiquitination and signaling [41]. The USP18 deficiency promotes TCR/CD28-stimulated activation of the TAK1 downstream kinases IKK and JNK as well as the transcription factors NF-κB and NFAT, resulting in hyper induction of genes encoding IL-2 and IFNγ. As will be discussed in the following section, USP18 also plays an important role in regulating CD4^+^ T cell differentiation.

A20 is another DUB that negatively regulates the NF-κB signaling pathway as well as other inflammatory pathways [42] (Fig. 2). Although A20 has been most extensively studied in innate immune cells, emerging evidence suggests that this DUB also plays an important role in the regulation of T cell activation and survival. A20 has an important role in regulating CD8 T cell responses [43]. This function of A20 involves inhibition of NF-κB signaling, and A20 deletion in mature T cells causes hyper production of IL-2 and IFNγ in CD8^+^ T cells through increased NF-κB activation. High levels of A20 expression in tumor-infiltrating CD8^+^ T cells are associated with poor anti-tumor immunity, and deletion of A20 increases the capability of CD8 T cells to reject tumors [43]. Another study suggests that A20 has opposing roles in the regulation of primary and memory responses of CD8^+^ T cells [44]. Mice with T cell-specific A20 deletion mount stronger immune responses during primary infection with *Listeria monocytogenes*. However, these mutant animals have impaired secondary immune responses against *Listeria* reinfection due to profound loss of pathogen-specific effector and memory CD8^+^ T cells [44]. A20 appears to inhibit the expression of the death receptor Fas (also called CD95) and prevent Fas-induced CD8^+^ T cell apoptosis [44]. A20 also plays a crucial role in regulating the survival of activated CD4^+^ T cells, which involves deconjugation of ubiquitin chains from K5 of RIPK3 [45]. The K5 ubiquitination of RIPK3 serves as a trigger for formation of RIPK1-RIPK3 complexes that are required for the induction of necroptotic cell death [45]. Thus, A20 deficiency promotes RIPK3 ubiquitination and formation of the RIPK1-RIPK3 complexes, causing exacerbated CD4^+^ T cell death [45]. Consistently, RIPK3 deficiency restores the survival of A20-deficient T cells and partially rescues the perinatal death of A20-KO mice [45]. Another mechanism of A20-mediated T cell survival is through regulation of autophagy [46]. A20 promotes autophagy in CD4^+^ T cells by inhibiting the activation of mTOR complex 1 (mTORC1), a kinase that serves as a major inhibitor of autophagy [46]. Consistent with an earlier study that TRAF6-mediated K63 ubiquitination of mTOR triggers its activation [47], A20 inhibits mTOR through deconjugating its polyubiquitin chains [46].

While several DUBs negatively regulate TCR-stimulated NF-κB signaling, the DUB USP9X serves as a positive regulator of this pathway [48]. USP9X physically interacts with Bcl10 in the CBM complex and inhibits TCR-stimulated Bcl10 ubiquitination. USP9X appears to remove K48-linked ubiquitin chains from Bcl10. Interestingly, however, USP9X knockdown does not promote Bcl10 degradation despite its increased K48 ubiquitination. The ubiquitination of Bcl10 seems to interfere with its association with CARMA1 and MALT1 [48].

The NFAT signaling pathway is also subject to ubiquitin-dependent regulation. Recent studies demonstrate that the activated form of NFATc2 is conjugated with K48 ubiquitin chains by the E3 ubiquitin ligase MDM2 and targeted for proteasomal degradation [49] (Fig. 2). Pharmacological inhibition or genetic deletion of MDM2 enhances nuclear NFATc2 along with T cell activation, which is associated with hyper induction of cytokines, including IL-2 and IFNγ. Interestingly, this negative mechanism of NFAT regulation also requires a DUB, USP15, which functions by stabilizing MDM2. Along with TCR/CD28 stimulation, MDM2 is transiently downregulated due to ubiquitin-dependent degradation, and the MDM2 degradation is greatly accelerated in USP15-deficient T cells. USP15 physically interacts with MDM2 and inhibits the ubiquitination and degradation of MDM2. Thus, USP15 can be considered a partner of MDM2 in the regulation of NFAT ubiquitination and T cell activation (Fig. 2). Since USP15 also stabilizes MDM2 in cancer cells, in which MDM2 serves as a major survival factor, ablation of USP15 appears to inhibit tumor growth by both promoting anti-tumor T cell responses and causing tumor cell apoptosis [49].

Ubiquitination also regulates the TCR proximal signaling events [24]. Compared to the extensive studies of E3 ubiquitin ligases, much less is known regarding the DUBs that are involved in the regulation of TCR-proximal signaling. A recent study has identified an OTU family of DUB, Otud7b (also called Cezanne), as a pivotal regulator of Zap70 activation [50] (Fig. 2). In response to TCR/CD28 stimulation, Otud7b is rapidly recruited to Zap70, and this molecular event is associated with Zap70 ubiquitination and requires the UBA domain of Otud7b. Thus, the Zap70/Otud7b interaction is likely facilitated by the ubiquitin binding function of Otud7b. Zap70 ubiquitination is known to promote its binding and dephosphorylation by two homologous tyrosine phosphatases, Sts1 (also called TULA-2 or Ubash3b) and Sts2 (also called TULA or Ubash3a) [51,52]. Consistently, the Otud7b-mediated Zap70 deubiquitination inhibits Zap70 interaction with Sts1 and Sts2 and promotes TCR/CD28-stimulated Zap70 phosphorylation and downstream signaling events [50]. Another DUB that has been shown to positively regulate TCR-proximal signaling and T cell activation is USP9X [53]. Although USP9X is dispensable for TCR-stimulated phosphorylation of Zap70, the USP9X deficiency attenuates phosphorylation of Zap70 target proteins, LAT, PLC-g1, Vav, and SLP76 [53], which is likely due to sequestration of Zap70 in the early endosomes [54]. USP9X appears to prevent endosomal sorting of Zap70 by inhibiting Zap70 monoubiquitination (Fig. 2). Unlike Otud7b, USP9X does not directly interact with Zap70 but rather comes into the proximity of Zap70 via interaction with a LAT signaling complex [54]. Interestingly, despite the positive role of USP9X in regulating TCR signaling, mice carrying T cell-specific deletion of USP9X display spontaneous T cell activation and develop a lupus-like autoimmune disease [53]. This seemingly controversial result is likely due to a defect of the USP9X-deficient mice in thymocyte negative selection and possible generation of self-reactive T cells [53]. A more recent study suggests that USP9X also deconjugates K48-linked polyubiquitin chains from Themis, a TCR-proximal signaling molecule regulating thymocyte development [55]. These findings, along with the earlier report that USP9X regulates CBM signaling complex and NF-κB activation [48], emphasize important roles for USP9X in regulating TCR signaling in both developing thymocytes and peripheral T cells and suggest the involvement of different targets of USP9X.

### DUBs in CD4^+^ T cell differentiation

CD4^+^ T cell differentiation is regulated by both the TCR signals and cytokine milieu [56]. TCR signal strength is thought to play an important role in the fate decisions toward different subsets of effector T cells. It is generally believed that strong and weak TCR signals favor CD4^+^ T cell differentiation toward Th1 and Th2 subsets, respectively [56]. Similarly, the TCR signaling strength also regulates the fate balance tween Th17 and iTreg cells, with stronger TCR signals favoring the differentiation of Th17 cells and weaker TCR signals favoring differentiation of iTreg cells. A recent study suggests that the TCR strength also determines T cell activation versus quiescence through influencing the TGFβ receptor I (TβRI) expression [57]. Strong TCR stimulation downregulates TGFβ receptor I expression via activation of the CARMA1/NF-κB pathway and, thereby, promotes T cell activation. It remains to be determined whether this mechanism of TβRI regulation also plays a role in T cell differentiation. Innate immune cells, particularly DCs, are also pivotal regulators of CD4^+^ T cell differentiation [58]. In addition to serving as APCs, DCs secrete different cytokines in response to different types of pathogens, which in turn guides the differentiation of CD4^+^ T cells to the generation of efficient effector cells required for pathogen clearance. Ubiquitination regulates T cell differentiation through both cell-intrinsic mechanisms involving control of TCR/CD28 signaling and cell-extrinsic mechanisms by regulating cytokine production in innate immune cells [24].

### Th1 differentiation

**Fig. 3:**
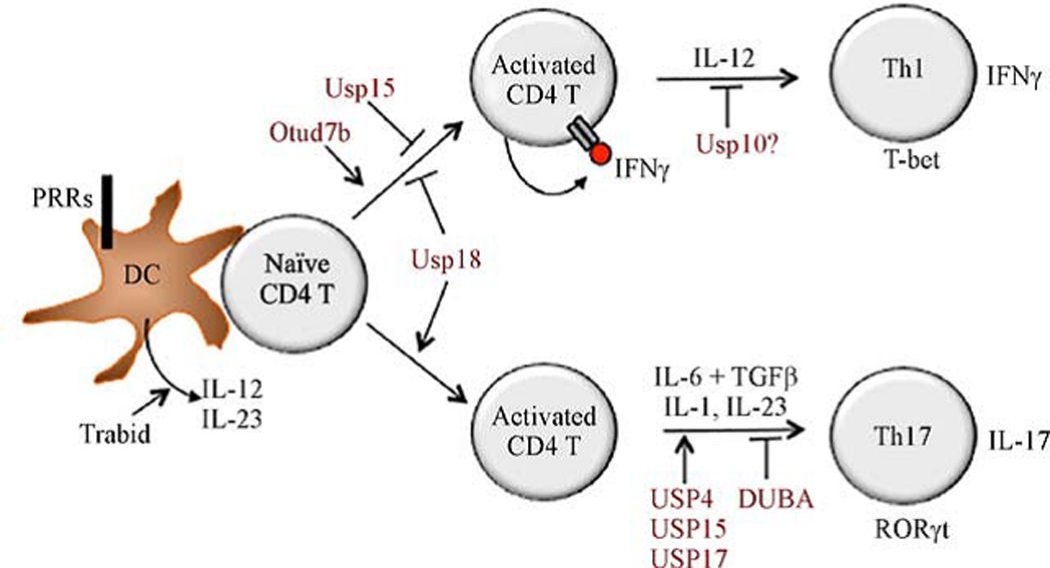
DUBs involved in regulation of CD4^+^ T cell differentiation. DUBs may regulate CD4^+^ T cell differentiation through controlling cytokine production during the early phase of T cell activation or regulating the lineage transcription factors during the subsequent phase of differentiation. In addition to the polarizing cytokine IL-12, IFNγ produced during T cell activation promotes Th1 differentiation. USP15 and USP18 attenuate Th1 differentiation by negatively regulating IFNγ induction, whereas Otud7b has the opposite function. USP18 promotes Th17 cell differentiation by inhibiting production of the Th17-inhibitory cytokine IL-2. Several DUBs (USP4, USP15, and USP17) promote Th17 polarization by stabilizing or facilitating the function of RORγτ, whereas DUBA inhibits Th17 polarization by promoting RORγτ degradation. The DUB Trabid promotes Th1 and Th17 cell differentiation and inflammation by facilitating TLR-induced expression of the polarizing cytokines IL-12 and IL-23.

Th1 cells are characterized by production of the cytokine IFNγ and participation of immune responses against intracellular pathogens. IL-12 produced by DCs and other innate immune cells serves as a major polarizing cytokine for Th1 differentiation [56]. In addition, IFNγ produced during the early phase of T cell activation promotes Th1 responses by inducing the expression of the Th1 lineage transcription factor T-bet [59]. The early phase production of IFNγ in activated CD4^+^ T cells is subject to regulation by the DUB USP15 [49]. USP15 deficiency renders CD4^+^ naïve T cells hyper-responsive to TCR/CD28 stimulation for IFNγ production, which is associated with enhanced Th1 differentiation *in vitro* in the presence of suboptimal doses of the Th1-polarizing cytokine IL-12 [49] (Fig. 3). Furthermore, the USP15-deficient mice display enhanced Th1 responses *in vivo* in both bacterial infection and tumorigenesis models [49]. Mechanistically, USP15 controls the fate of NFATc2, a transcription factor critically involved in the induction of IFNγ. USP15 functions as a DUB of the E3 ubiquitin ligase MDM2, which in turn mediates K48 ubiquitination and proteolysis of activated NFATc2. Along with T cell activation, MDM2 itself is targeted for ubiquitin-dependent degradation, a process protected by USP15-mediated MDM2 deubiquitination. In the absence of USP15, MDM2 is rapidly degraded upon TCR/CD28 stimulation, which is associated with elevated levels of nuclear NFATc2 and hyper-induction of IFNγ and IL-2 [49]. In contrast to the negative role of USP15 in Th1 responses, the OTU family DUB Otud7b positively regulates Th1 differentiation under both *in vivo* and *in vitro* conditions [50]. Otud7b appears to promote Th1 responses by facilitating TCR-proximal signaling and early induction of IFNγ along with T cell activation [50] (Fig. 3). Ubiquitination may also regulate Th1 responses through controlling the stability of Th1-polarizing transcription factors. An *in vitro* study suggests that USP10 physically interacts with and inhibits the ubiquitination and degradation of T-bet [60] (Fig. 3). However, it remains to be examined whether USP10 plays a role in regulating Th1 cell differentiation under *in vitro* and *in vivo* conditions.

### Th17 differentiation

USP18 is a DUB that positively regulates Th17 cell differentiation and the pathogenesis of the autoimmune disease EAE [41]. As discussed earlier, USP18 negatively regulates the TAK1/IKK signaling pathway stimulated by the TCR/CD28 signals. Thus, like the USP15-deficient T cells, the USP18-deficient T cells are sensitized for Th1 generation likely due to hyper-production of IFNγ during the activation phase [41] (Fig. 3). However, under Th17 differentiation conditions, the USP18 deficiency attenuates CD4^+^ T cell differentiation into Th17 cells. This is at least partially due to IL-2 overproduction by the activated USP18-deficient CD4^+^ T cells, since IL-2 neutralization partially rescues the defect of the USP18-deficient CD4^+^ T cells in Th17 cell differentiation [41].

Another Th17-regulatory DUB is DUBA (also named OTUD5) [61], which was originally identified as a DUB of TRAF3 regulating type I IFN induction in macrophages [62]. T cell-specific deletion of DUBA promotes Th17 cell production both *in vitro* and *in vivo* and renders mice hyper-sensitive to the induction of intestinal inflammation by injection with a TCR-agonistic antibody, anti-CD3 [61]. The DUBA deficiency also renders Treg cells producing IL-17A upon TCR stimulation, although these DUBA-deficient Treg cells still retain immunosuppressive functions both *in vitro* and *in vivo*. Consistently, the T cell-conditional DUBA knockout mice do not develop autoimmunity even at an older age (1 year) [61]. Mechanistically, DUBA interacts with and stabilizes UBR5, an E3 ubiquitin ligase mediating ubiquitination and degradation of the Th17 lineage transcription factor RORγτ. Thus, DUBA knockout or UBR5 silencing enhances the level of RORγτ and promotes differentiation of Th17 cells [61]. In addition to its role in regulating CD4^+^ T cell differentiation, DUBA also plays a role in regulating thymocyte development, since T cell-specific deletion of DUBA reduces the number of CD4^+^ and CD8^+^ mature thymocytes [61].

*In vitro* studies suggest the involvement of several other DUBs, including USP4, USP15, and USP17, in the regulation of Th17 cell differentiation [63–65] (Fig. 3). USP4 and USP17 stabilize RORγτ by deconjugating K48-linked polyubiquitin chains from RORγτ, thereby promoting Th17 differentiation. shRNA-mediated knockdown of USP4 or USP17 reduces the level of RORγτ and attenuates the expression of IL-17 and other Th17-related genes [63,64]. USP15 also targets RORγτ for deubiquitination, but USP15 regulates the function, instead of stability, of RORγτ [65]. USP15 removes ubiquitin from lysine 446 of RORγτ, which facilitates the association of RORγτ with steroid receptor coactivator 1 (SRC1), thereby promoting the transactivation function of RORγτ and Th17 differentiation [65]. However, an *in vivo* study using USP15 knockout mice did not reveal reduced production of IL-17 in a model of chemical-induced fibrosarcoma [66]. It remains to be examined whether USP15 regulates Th17 cell differentiation in autoimmunity models, such as EAE, and whether USP4 and USP17 function *in vivo* in Th17 responses. It is also interesting to examine how USP4 and USP17 function non-redundantly in the regulation of RORγτ ubiquitination and proteolysis.

### Cell-extrinsic mechanism of Th cell regulation

DUBs also regulate CD4^+^ T cell differentiation through modulation of cytokine expression in innate immune cells. One such DUB is Zranb1 (also called Trabid), which is specifically required for induction of IL-12 and IL-23 proinflammatory cytokines in dendritic cells [67]. Zranb1 mediates TLR-stimulated histone modifications at the promoters of Il12/Il23 genes (Il12a, Il12b, Il23a), which in turn regulates the recruitment of NF-κB members. This epigenetic mechanism involves Zranb1-mediated deubiquitination and stabilization of a histone demethylase, Jmjd2b, which is required for erasing the transcriptionally repressive histone marks H3K9me2 and H3K9me3 in the Il12/Il23 promoters [67]. A20 also plays an important role in DCs to indirectly regulate Th cell responses [68]. DC-conditional deletion of A20 causes spontaneous proliferation of T cells and their differentiation into IFNγ-producing effector T cells, which is associated with systemic autoimmunity [68]. In the intestine, the DC-conditional A20 knockout mice have enhanced frequency of inflammatory Th1 and Th17 cells, associated with lymphocyte-dependent colitis [69,70]. A20-silenced bone marrow-derived macrophages also promote the cytotoxicity of CD8^+^ and CD4^+^ T cells *in vitro* [71].

### DUBs in T cell tolerance

The maintenance of T cell tolerance is mediated by both central and peripheral tolerance mechanisms. Central tolerance occurs along with thymocyte development and involves deletion or functional inactivation of self-reacting T cells by negative selection [4]. Ubiquitination plays a crucial role in the regulation of central tolerance, and a number of E3 ubiquitin ligases have been characterized for this function [24]. In contrast, the DUBs that regulate T cell central tolerance have been poorly defined. A DUB implicated in the regulation of central tolerance is USP9X [53]. USP9X deficiency causes the expansion of antigen-experienced T cell populations and the development of an autoimmune and lymphoproliferative disease [53]. USP9X may regulate thymocyte negative selection for eliminating self-reactive T cells through mediating TCR-proximal signaling.

The peripheral mechanism of T cell tolerance involves anergy induction and Treg-mediated suppression of self-reactive T cells that have escaped from elimination during central tolerance [4,72]. Both the development and the immunosuppressive function of Treg cells are subject to regulation by ubiquitination. CYLD is a DUB that negatively regulates the development of Treg cells, and the CYLD deficiency enhances Treg frequency in both the thymus and peripheral lymphoid organs [73]. The role of CYLD in regulating Treg development seems to involve inhibition of NF-κB signaling pathway, since NF-κB is a strong inducer of Treg development [74]. In addition, CYLD may also regulate Treg development via inhibition of TGFβ signaling, in which CYLD deubiquitinates Smad7 and, thereby, inhibits activation of TAK1 and p38 [75]. Enhanced Treg production has also been found in mice expressing a nonfunctional CYLD splice variant, CYLD(ex7/8) [76]. Interestingly, the Treg cells from these mutant mice display impaired suppressive function. This latter study suggests that although CYLD inhibits Treg cell development, it positively regulates the immunosuppressive function of Treg cells. It remains to be examined whether complete deletion of CYLD also impairs the function of Treg cells. Like CYLD, A20 negatively regulates thymic development of Treg cells, which involves inhibition of the canonical NF-κB member RelA [77]. T cell-specific deletion of A20 causes an increase in the number of Treg cells in both the thymus and peripheral lymphoid organs. The loss of A20 does not promote the proliferation or survival of Treg cells but appears to render thymic Treg precursor cells less dependent on IL-2 for *in vivo* development [77]. A20-deficient Treg cells retain immunosuppressive functions.

The role of ubiquitination in regulating the stability and function of established Treg cells was demonstrated by a study using mutant mice deficient in Ubc13, an E2 ubiquitin-conjugating enzyme that functions together with Uev1A as a heterodimer to specifically conjugate K63-linked ubiquitin chains [78]. Treg-specific deletion of Ubc13 in mice using Foxp3-Cre impairs the immunosuppressive function of Treg cells *in vivo* and renders them sensitive to acquisition of inflammatory Th1- and Th17- like phenotypes, which is associated with aberrant T cell activation and development of autoimmunity [78]. This function of Ubc13 involves ubiquitin-dependent activation of the IKK/NF-κB signaling pathway in Treg cells, which in turn mediates transcriptional induction of an anti-inflammatory factor SOCS1 that suppresses proinflammatory cytokine signaling [78]. It remains to be examined whether the Ubc13-opposing DUBs, such as CYLD and A20, play a role in regulating the function of established Treg cells.

Ubiquitination also regulates Treg function through targeting Foxp3, a master transcription factor of Treg cells. K48 ubiquitination of Foxp3 by the E3 ubiquitin ligase Stub1 targets Foxp3 for proteasomal degradation, whereas the DUB USP7 stabilizes Foxp3 by deconjugating its ubiquitin chains [79,80]. Genetic ablation or pharmacological inhibition of USP7 reduces the level of Foxp3 protein and impairs the immunosuppressive function of Treg cells [80,81]. USP7 also deubiquitinates and stabilizes Tip60 [81], a histone acetyltransferase known to acetylate and activate Foxp3 for maintaining Treg stability and immune tolerance [82]. USP7 deficiency results in ubiquitin-dependent degradation of Tip60, which contributes to the instability and functional inactivation of Foxp3 and impaired Treg function [81]. A selective inhibitor of USP7, USP7i, inhibits the function of Treg cells, without affecting conventional T cells, and has been shown to promote antitumor immunity [81]. Recent studies have elucidated the structural basis of USP7 inhibition by small-molecule inhibitors [83,84], providing additional insight for developing USP7-based therapeutic drugs.

DUBs also regulate T cell tolerance in an indirect manner. For example, A20 functions in DCs to suppress DC activation and maintain tolerance of self-reactive T cells [68]. A20 deficiency in DCs promotes induction of survival genes by CD40 ligand and RANK and enhances the ability of DCs to capture apoptotic cells and to induce the generation of self-reactive Th1 and Th17 inflammatory T cells. Consequently, the DC-conditional A20 knockout mice develop systemic autoimmunity, characterized by increased serum concentrations of autoantibodies and recurrent abortion in female pregnant mice similar to anti-phospholipid syndrome (APLS) [68].

### Concluding remarks

Recent progress has revealed crucial roles of DUBs in the regulation of different aspects of T cell functions, ranging from T cell development, activation, and differentiation to T cell homeostasis and tolerance, implicating DUBs as potential therapeutic targets for the treatment of immunological disorders and cancer. However, despite the extensive progress in this field, many missing links exist. For example, the molecular mechanism underlying the action of different DUBs remains unclear, and how the function of specific DUBs is regulated in T cells under homeostatic and antigen-stimulated conditions is poorly understood. The primary substrates for most T cell-regulatory DUBs are also incompletely defined. Another missing link is how DUBs dynamically act to oppose the function of specific E3s. Recently developed techniques to detect *in vivo* protein–protein interactions will allow the determination of DUB-associated signaling complexes. Moreover, the development of DUB inhibitors with high selectivity represents an important future direction that is important for both therapeutic applications and further investigation of DUB functions in T cells.

## Figures and Tables

**Table 1:** Deubiquitinases involved in T cell regulation

DUB	Family	Function	Target	References

CYLD	USP	Thymocyte development	LCK, IKK	[25, 27]
		Survival of immature NKT cells	IKK	[29]
		T cell activation	TAK1, IKK	[39, 40]
		Treg development	IKK, Smad7	[73, 75, 76]
USP4	USP	Th17 differentiation	RORγτ	[64]
USP7	USP	Treg function	Foxp3, Tip60	[79–81]
USP8	USP	Thymocyte maturation	CHMP5	[35, 36]
USP9X	USP	TCR signaling	Bcl10	[48]
		TCR signaling and central tolerance	Zap70	[53, 54]
		TCR signaling	Themis	[55]
USP10	USP	Unknown	T-bet	[60]
USP15	USP	T cell activation and differentiation	MDM2	[49]
		Th17 differentiation	RORγτ	[65]
USP17	USP	Th17 differentiation	RORγτ	[63]
USP18	USP	Th17 differentiation	TAK1-TAB1	[41]
A20	OTU	NKT cell differentiation	MALT1	[33]
		CD8 T cell activation	NF-κB pathway	[43, 44]
		CD4 T cell survival	RIPK3	[45]
		T cell survival	mTORC1	[46]
		Cell-extrinsic regulation of Th1 and Th17 cell differentiation	NF-κB	[68–70]
		Treg development	pathway NF-κB pathway	[77]
Otud7b	OTU	T cell activation and differentiation	Zap70	[50]
DUBA	OTU	Th17 differentiation	UBR5	[61]
Zranb1	OTU	Cell-extrinsic regulation of Th1 and Th17 cell differentiation	Jmjd2b	[67]
